# Chiropractic and exercise for seniors with low back pain or neck pain: the design of two randomized clinical trials

**DOI:** 10.1186/1471-2474-8-94

**Published:** 2007-09-18

**Authors:** Michele J Maiers, Jan Hartvigsen, Craig Schulz, Karen Schulz, Roni L Evans, Gert Bronfort

**Affiliations:** 1 Northwestern Health Sciences University, Wolfe-Harris Center for Clinical Studies, 2501 West 84^th ^Street, Bloomington, MN 55431, USA; 2 University of Southern Denmark, Campusvej 55, DK-5230 Odense M, Denmark; 3 Nordic Institute of Chiropractic and Clinical Biomechanics, Clinical Locomotion Science, Forskerparken 10, 5230 Odense M, Denmark

## Abstract

**Background:**

Low back pain (LBP) and neck pain (NP) are common conditions in old age, leading to impaired functional ability and decreased independence. Manual and exercise therapies are common and effective therapies for the general LBP and NP populations. However, these treatments have not been adequately researched in older LBP and NP sufferers.

The primary aim of these studies is to assess the relative clinical effectiveness of 1) manual treatment plus home exercise, 2) supervised rehabilitative exercise plus home exercise, and 3) home exercise alone, in terms of patient-rated pain, for senior LBP and NP patients. Secondary aims are to compare the three treatment approaches in regards to patient-rated disability, general health status, satisfaction, improvement and medication use, as well as objective outcomes of spinal motion, trunk strength and endurance, and functional ability. Cost-effectiveness and cost-utility will also be assessed. Finally, using qualitative methods, older LBP and NP patient's perceptions of treatment will be explored and described.

**Methods/Design:**

This paper describes the design of two multi-methods clinical studies focusing on elderly patients with non-acute LBP and NP. Each study includes a randomized clinical trial (RCT), a cost-effectiveness study alongside the RCT, and a qualitative study. Four hundred and eighty participants (240 per study), ages 65 and older, will be recruited and randomized to one of three, 12-week treatment programs. Patient-rated outcome measures are collected via self-report questionnaires at baseline and at 4, 12, 26, and 52 weeks post-randomization. Objective outcomes are assessed by examiners masked to treatment assignment at baseline and 12 weeks. Health care cost data is collected through standardized clinician forms, monthly phone interviews, and self-report questionnaires throughout the study. Qualitative interviews using a semi-structured format are conducted at the end of the 12 week treatment period.

**Discussion:**

To our knowledge, these are the first randomized clinical trials to comprehensively address clinical effectiveness, cost-effectiveness, and patients' perceptions of commonly used treatments for elderly LBP and NP sufferers.

**Trial Registration:**

NCT00269321 and NCT00269308

## Background

Low back pain (LBP) and neck pain (NP) are major public health problems throughout the western world. These conditions can begin early in life [1,2] and persist through adulthood [3,4] and into old age [5]. This places LBP and NP among the most common health complaints experienced over a lifetime. Most research pertaining to LBP and NP has been aimed at the working and middle aged segments of the population. However, it is estimated that, by the year 2025, approximately one third of individuals in developed countries will be over 60 years of age[6]. Anticipating the impact of population projections, interest in LBP and NP among seniors has increased.

LBP and NP, either alone or in conjunction, affect over 30% of the population 70 years of age and older on a monthly basis [5]. These conditions have important impact, since approximately 15% of this population indicate that they have subsequently altered or diminished their physical activity during the past year due to LBP or NP. Roughly the same proportion have sought some kind of treatment [7]. Furthermore, LBP has been rated as the third most important condition affecting the physical health status of older Americans, after heart and lung disease [8]. NP has also been found to substantially impact function and well-being in this age group [9]. Thus, while LBP and NP are not life threatening conditions, they may lead to reduced functional ability and decreased independence, resulting in serious socio-economic consequences for elderly individuals, their families, and society [10]. Therefore, research aimed at identifying effective prevention and treatment strategies is a high priority.

Spinal manipulative therapy (SMT) is one of the most commonly used treatment modalities for spinal pain in both younger and older persons [11,12]. Authors of recent systematic reviews conclude that the effect of SMT is similar to that of other commonly used forms of treatment for many types of LBP and NP [13-15]. To our knowledge, no randomized clinical trials comparing the effect of SMT with other forms of treatment for LBP and NP in older persons have been conducted.

Exercise is a commonly prescribed treatment for LBP and NP. An active lifestyle involving regular strenuous physical activity has been found to protect against the incidence of LBP among older persons [16]. Additionally, a systematic review of 61 randomized clinical trials by Hayden et al found exercise to be effective in reducing pain and improving function in persons with chronic LBP [17]. They also noted that individualized exercise was more effective if supervised [18]. However, there is a paucity of clinical trials involving elderly patients and it is unknown if these findings also apply to this age group.

Minimal intervention in the form of home exercises and self-care is also commonly used in LBP and NP management [19], and has been shown in some controlled trials to be as effective as more aggressive and more costly alternatives [20,21]. As such, self-care is an attractive control group in randomized clinical trials, where it represents a credible alternative to placebo or wait-list, thereby enhancing patient compliance [22,23]. It is also an attractive treatment option in clinical practice, representing an easy and cost-effective way of managing a common and costly problem. A trial by Haas et al [24] compared a self-care program, designed to address chronic pain conditions to a wait-list, among seniors with LBP. The authors found no advantage to self-care over the wait-list in terms of self-efficacy, pain, or general health. These findings may be due to the non-specificity of the self-care program; further studies of seniors are needed to assess self-care that specifically addresses LBP and NP among this group.

In summary, on-going LBP and NP have substantial impact on the functional capacity and well-being of older people, in the absence of effective prevention and treatment strategies. We, therefore, designed two parallel multi-methods clinical studies focusing on elderly patients with non-acute LBP and NP. Each study includes a randomized clinical trial (RCT), a cost-effectiveness study alongside the RCT, and a qualitative study. The primary aims of the RCT are to determine the relative clinical effectiveness of 1) chiropractic manual therapy plus home exercise, 2) supervised rehabilitative exercises and home exercise, and 3) home exercise alone for LBP and NP patients 65 and older in both the short-term (12 weeks) and long-term (one year) using pain as the primary outcome measure. Secondary aims are to assess the short- and long-term relative effectiveness of the three interventions, using 1) patient-rated outcomes regarding back and neck disability, general health status, patient satisfaction, improvement, and medication use; 2) objective functional performance outcomes of spinal motion, trunk strength and endurance, and functional ability; and 3) cost-effectiveness and cost utility measures. Finally, the qualitative studies will describe LBP and NP patients' perceptions of treatment and the issues they consider when determining their satisfaction with care.

## Methods/Design

These randomized clinical trials are being conducted at the Wolfe-Harris Center for Clinical Studies at Northwestern Health Sciences University in Minneapolis, Minnesota. They began in 2003, and are ongoing. Approval has been granted by the institutional review boards of all participating institutions, and informed consent is obtained from all participants.

### Study population

Four hundred and eighty participants (240 per study) are being recruited from the Twin Cities metropolitan area through the use of newspaper advertising, targeted postcard mailings, community outreach, and an existing registry generated from previous trials conducted by the researchers.

### Inclusion criteria

Participants in these studies must be 65 years of age or older, independently ambulatory, community dwelling, on a stable pain medication plan (if medications are taken), and score a minimum of 20 points on the Folstein Mini-Mental State Examination [25].

The studies focus on individuals with a primary complaint of mechanical low back or neck pain, which has no specific, identifiable etiology but can be reproduced by movement or provocation tests. Eligible patients must have a clinical presentation that meets the Quebec Task Force categories of 1, 2, 3, or 4, which includes individuals with neck or back pain, stiffness or tenderness, with or without radiation or neurological signs [26]. The back pain study includes individuals with sub-acute and chronic LBP lasting a minimum of 6 weeks in duration; the neck pain study focuses on chronic NP 12 or more weeks in duration.

### Exclusion criteria

Participants are excluded if they have a baseline pain score of less than 30 percentage points, pain referred from the joints of the extremities or visceral diseases, suffer from significant infectious disease, are currently receiving ongoing treatment for low back or neck pain, or have any contraindications to exercise or spinal manipulation.

### Eligibility Determination

Interested individuals contact the research center by phone or mail. Trained study staff contact these individuals by phone and administer a short questionnaire to determine obvious inclusion and exclusion criteria. Data is directly entered into a relational database that immediately determines eligibility for baseline evaluation. Eligible patients are scheduled for a baseline evaluation, which includes informed consent, a self-report questionnaire, health history and physical examination, x-rays, and blinded, objective assessment.

All potential participants have their case reviewed by a group of chiropractic and allopathic study clinicians during weekly "case review" meetings, which facilitates consistent interpretation and application of the pre-defined eligibility criteria. Participants who satisfy the inclusion criteria attend a second baseline evaluation, which includes review of the study, a self-report questionnaire, and blinded, objective outcomes assessment. If all eligibility requirements are satisfied, participants are then randomly assigned to one of the three interventions.

### Randomization

Restricted randomization with a 1:1:1 allocation ratio has been applied using randomly permuted block sizes. The randomization scheme and block sizes are concealed from the study team to ensure they are masked to the sequence of treatment assignments. As individuals become eligible, sequentially numbered opaque envelopes with treatment assignments are drawn.

### Treatments

All participants in the study receive 12 weeks of care in one of three treatment groups. They are asked not to seek any additional treatment for their neck or back pain during the treatment period. Treatments are documented on standardized forms, and care providers, trained in study protocols, are monitored for compliance through chart audit, observations, and team meetings.

### Home exercise program

Participants in the home exercise program (HEP) attend four, 45–60 minute sessions with an exercise therapist. At the first two sessions, participants are given simple information about how to manage their neck or back pain. This includes postural instructions and practical demonstrations of proper body mechanics for lifting, pushing, pulling, and rising from a lying position, all performed with patient participation. They are also given information on self-care for pain management, including the use of ice, heat, and medication. Importantly, patients are reassured that movement and exercise are good for their back and neck, even if they experience some discomfort or have an arthritic condition. To reinforce the message to stay active, patients are given instructions to perform specific exercises designed to improve balance and coordination, as well as enhance trunk strength and endurance without excessive loading [27].

Exercises in both programs are tailored to the individual patient's level of ability and are executed on a graded progression over 12 weeks. The LBP program includes the following exercises:

• Stretching: seated or standing lumbar flexion, full spine flexion/extension motion cycles, quadriceps stretch, hamstring stretch, hip stretch, head retraction, and chest expansion.

• Muscle Strength and Endurance: chair squats, abdominal curls, seated back extension (isometric or using resistance tubing), seated upright rows (using resistance tubing), and push ups.

• Balance: standing knee lifts, standing straight-leg hip flexion and extension.

The NP program consists of the following:

• Stretching: head retraction, chest expansion, full spine flexion/extension motion cycles, hamstring stretch, quadriceps stretch, and hip stretch.

• Endurance: cervical flexion and extension (isometric or using resistance tubing), push ups, chest press (using resistance tubing), seated upright rows (using resistance tubing), chair squats, and abdominal curls.

• Balance: standing knee lifts, standing straight-leg hip flexion and extension.

Participants are encouraged to perform the stretching exercises daily, and the strength and balance exercises 3–4 days per week in their home. They are also given a binder with handouts of written and illustrated descriptions of each exercise, and a simple diary to record their exercise progress. The last two sessions give study participants the opportunity to ask questions and perform the exercises with the therapist who can suggest progressions and ensure correct form.

### Chiropractic manual treatment plus home exercise

Participants allocated to this group receive chiropractic manual treatment in addition to the home exercise program (described above).

Manual treatment is delivered by a chiropractor, who uses pain provocation [28] and static/motion palpation [29] findings to determine areas of treatment. Care may include spinal manipulation, mobilization and flexion-distraction therapy, with light soft tissue massage as indicated to facilitate the manual therapy [30]. The type of manual treatment technique and the force applied to the spinal structures are modified to accommodate the age and physical condition of the study participant. The number and frequency of treatments is determined by the individual chiropractor, with a maximum of 20 visits.

### Supervised rehabilitative exercise plus home exercise

Participants assigned to this group participate in a supervised rehabilitative program in addition to the home exercise program (described above).

Rehabilitative exercise consists of 20, 1-hour sessions supervised by an exercise therapist. Emphasis is placed on performing high repetitions of low load exercises with the aim of increasing endurance, strength, and balance. Each session begins with a light aerobic warm up, consisting of 10–15 minutes on a stationary bicycle, treadmill, or elliptical trainer. Exercises focus on stretching, strength, endurance and balance, similar to the HEP. The LBP program also includes neck flexion, quadruped, lunges, side bridging, and trunk extension exercises on an adjustable angle roman chair. The NP program additionally includes neck flexion, shoulder shrugs, and trunk extension exercises on an adjustable angle roman chair. Both the LBP and NP supervised exercise programs take place under the individualized guidance of exercise therapists who closely monitor form, modify exercises, prescribe progressions, and provide encouragement.

### Outcome measures

**Figure 1:**
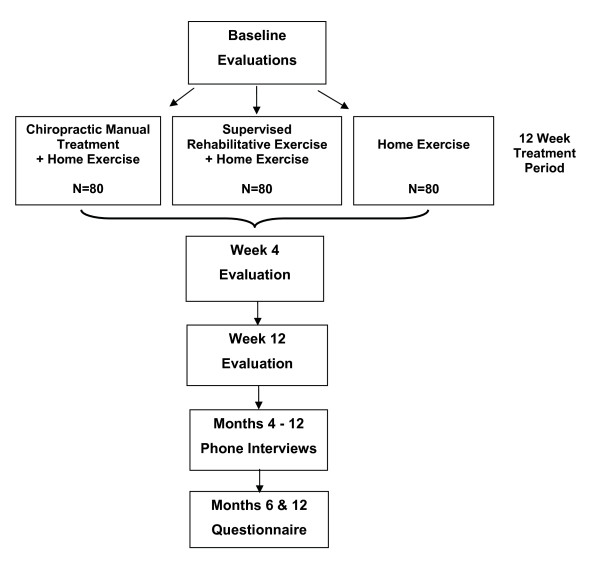
Participant flow, study visits, and evaluations.

The outcomes chosen for these studies are measured by patient self-report, blinded objective assessment, and in-person and telephone interviews. They are collected at baseline, during the 12-week treatment period, and over the course of one year following randomization. Participant flow, study visits, and evaluations are outlined in Figure 1, and are the same for both the LBP and NP studies.

### Patient self-report outcome measures

Patient self-report questionnaires are collected at baseline, and 4, 12, 26, and 52 weeks post-randomization.

#### Primary outcome measure

Participants are asked to rate their typical level of neck or back pain over the last week, using an ordinal 11-box scale (0 = no pain, 10 = the worst pain possible) [31].

#### Secondary outcome measures

• Disability is measured with the 23-item Modified Roland Scale [32,33] in the back pain study, and the Neck Disability Index[34] in the neck pain study.

• General health status is measured in both studies by the Medical Outcomes Study Short Form 36-item Health Survey (SF-36 D) [35].

• Improvement is rated by participants after 4 and 12 weeks of treatment on a 9-point ordinal scale, with responses ranging from "100% improved" to "100% worse" [36].

• Satisfaction with seven aspects of patient care, including information on diagnosis, prognosis, activities to hasten recovery, and prevention, as well as concern shown by providers, the quality of treatment recommendations, and overall care, are rated on a 5-point scale, with responses ranging from "poor" to "excellent" [37].

• Non-prescription and prescription medication use for LBP or NP is measured in days over the previous week [38,39].

### Objective outcome measures

Secondary objective outcomes are measured by study staff blinded to treatment assignment at baseline and week 12 (post-intervention).

#### Spinal biomechanical measures

• Lumbar and cervical spine dynamic motion are assessed using the Zebris CMS-HS Spine Motion Analyzer (Zebris Inc., Isny im Allgau, Germany) [40,41]. Participants are asked to perform flexion-extension, rotation, and lateral flexion, while data is collected on the range, velocity, and patterns of these motions.

• Isometric muscle flexion and extension strength is measured in the neutral position by a computerized load-cell transducer dynamometer (Promotron 3000, Promatek Medical Systems, Joilet, IL) for the cervical spine, and with a computerized digital myograph (DM2000, Myotech Corp., FL) for the lumbar spine.

• Static muscle endurance for the cervical spine is measured with participants holding their head in flexion (supine position) and extension (prone position) while holding 50% maximum voluntarily contraction resistance until muscle failure or to the limit of pain tolerance [38]. Static muscle endurance for the lumbar spine is measured with participants in a prone neutral position for extension and recumbent (60° angle) position for flexion. Participants hold this position until muscle failure or to the limit of pain tolerance [42,43].

#### General health and functional ability measures

• The "Timed Up and Go" test, which measures the time it takes to rise from a chair, walk 10 feet, turn around, and return to the chair, is used to assess basic functional ability of study participants [44,45].

• Hand grip strength is measured bilaterally with a hydraulic dynamometer (Jamar Hand dynamometer, Sammons Preston – U.S.A, Bolingbrook, IL) with the subjects positioned following the recommendations by the American Society of Hand Therapists [46].

### Health care costs/cost utility analysis

Direct costs for each patient will represent the one-year aggregated LBP or NP related health care costs based on utilization and estimated costs. Health care utilization (within and outside of the studies) is measured using standardized clinician treatment forms (each visit, weeks 1 to 12), monthly phone call interviews (weeks 16 to 52) and patient self-report questionnaires (baseline and weeks 4, 12, 26 and 52). Direct health care costs include costs related to study treatment, non-study health care health provider use, medication utilization, and hospitalizations for LBP or NP. Indirect costs of wage and productivity loss are measured through patient self-report (weeks 4, 12, 26, and 52) using three questions from the National Health Interview Survey (NHIS) that measure lost or impacted work or activity days due to back or neck pain [47]. The EuroQol 5D, a multi-attribute, patient self-report utility scale measuring five dimensions (mobility, self-care, usual activities, pain/discomfort, and anxiety/depression), is used as the cost-utility index [48,49]. It is measured at baseline and weeks 4, 12, 26, and 52.

### Qualitative interviews

Qualitative interviews are conducted on an individual basis at the end of the 12-week treatment period. The interviews are semi-structured with open-ended questions, asking how patients feel about the treatment they received, whether it meets their expectations, and what they like and dislike. Participants are also asked to identify the factors they consider when determining their satisfaction with care and which outcome measures they consider most important.

### Data analysis

#### Treatment effect

Power was calculated to detect a medium effect size difference in the primary outcome measure of patient self-report pain. A sample size of 240 subjects per study is needed to provide 88% power to detect a difference of eight percentage points between the highest and lowest group means, assuming and alpha level of 0.05. This allows for a 15% drop out or loss to follow-up rate.

Primary analysis will use repeated measures analysis of covariance (ANCOVA) to measure differences between the 3 groups in patient-rated pain using weeks 4 and 12 data for the short-term outcome, and weeks 4, 12, 26, and 52 for the long-term outcome. Baseline values will be used as covariates, and 95% confidence intervals will be placed on group differences. Possible treatment-time interactions will be accounted for and an intention-to-treat analysis will be used [50]. A confirmatory, secondary analysis using a repeated measures, multivariate analysis of covariance (MANCOVA) will be used as an overall test for differences between groups. This will include both the primary and the secondary patient-rated outcomes to assess short- and long-term efficacy.

#### Cost-effectiveness analysis

A cost comparison of the intervention and control groups will be performed using data on direct and indirect costs. Cost differences between groups will be estimated using regression analysis where all neck or back-related costs in a year are regressed on treatment. A cost-effectiveness analysis, using a mixed-model linear regression analysis, will be conducted to compare the interventions with home exercise (as the control), using patient-rated pain as the effectiveness measure. Finally, a cost-utility analysis comparing the intervention and control groups will be performed using the EuroQol-5D.

#### Qualitative analysis

Qualitative analysis of transcribed participant interviews will use an inductive approach toward content analysis, as a way of identifying themes and categories of responses [51-53]. Two investigators (MM and RE) will independently analyze the interviews and regularly meet to establish consensus on the coding of themes. Inter-rater reliability of text coding will be assessed, and kappa values of less than 0.8 will necessitate review of the coding structure. Categorized information from the transcribed interviews will be summarized and interpreted in the context of other study results. The frequency of themes will be quantified and representative patient quotations will be identified [53,54].

## Discussion

Low back pain (LBP) and neck pain (NP) are important health problems for both younger and geriatric individuals. Of particular concern is that conditions associated with LBP and NP, such as impaired strength and flexibility, can have very serious consequences for an older individual's independence and overall health. The best treatments for low back and neck conditions will not only aim to treat the pain specifically, but will also address associated strength and motion in a manner that enhances general function and improves quality of life. Chiropractic manual treatment and exercise are treatment approaches that aim to meet these needs and have demonstrated potential in younger individuals.

To our knowledge, these are the first randomized clinical trials to comprehensively address clinical effectiveness, cost-effectiveness, and patients' perceptions of commonly used treatments for elderly LBP and NP sufferers. This article presents the rationale and design of two mixed methods clinical trials, each consisting of an RCT, with cost-effectiveness and qualitative studies conducted alongside the central trial. Both are anticipated to be completed in 2007, at which time the results will be made available.

## Competing interests

The author(s) declare that they have no competing interests.

## Authors' contributions

The authors are investigators of the described clinical trials, and each contributed to study design. GB is the principal investigator. GB and RE are primarily responsible for the conceptualization of these trials. MM and JH prepared the first draft of the manuscript; MM coordinated subsequent revisions. JH made the primary contribution to the background and significance section of the proposal and this manuscript. CS and KS provided information for the methods section. All authors read and approved the final manuscript.

## Pre-publication history

The pre-publication history for this paper can be accessed here:


